# Ultra-High Frequency Distortion Product Otoacoustic Emissions for Detection of Hearing Loss and Tinnitus

**DOI:** 10.3390/ijerph19042123

**Published:** 2022-02-14

**Authors:** W. Wiktor Jedrzejczak, Edyta Pilka, Malgorzata Ganc, Krzysztof Kochanek, Henryk Skarzynski

**Affiliations:** 1 Department of Experimental Audiology, Institute of Physiology and Pathology of Hearing, Mochnackiego 10, 02-042 Warsaw, Poland; e.pilka@ifps.org.pl (E.P.); m.ganc@ifps.org.pl (M.G.); 2 World Hearing Center, ul. Mokra 17, Kajetany, 05-830 Nadarzyn, Poland; k.kochanek@ifps.org.pl (K.K.); skarzynski.henryk@ifps.org.pl (H.S.); 3 Institute of Physiology and Pathology of Hearing, Mochnackiego 10, 02-042 Warsaw, Poland; 4 Department of Oto-Rhino-Laryngosurgery, Institute of Physiology and Pathology of Hearing, Mochnackiego 10, 02-042 Warsaw, Poland

**Keywords:** tinnitus, otoacoustic emissions, distortion product otoacoustic emissions, DPOAE, pure tone audiometry

## Abstract

Several studies have suggested that distortion product otoacoustic emissions (DPOAEs) may be an early marker not only of hearing loss (HL) but also of tinnitus. The purpose of this study was to investigate whether DPOAEs measured up to 16 kHz are affected by the presence of tinnitus. Pure tone thresholds and DPOAEs were measured in two groups: 55 patients with tinnitus and 63 subjects without tinnitus. The subjects were divided into three groups according to their audiometric results—better than 25 dB HL at all tested frequencies from 0.125 to 16 kHz, better than 25 dB up to 8 kHz, and hearing impaired. Receiver operator characteristics (ROCs) were used to test whether DPOAEs could differentiate between normal hearing, hearing loss, and tinnitus. Comparison of tinnitus subjects with the control group, matched accurately according to thresholds, did not yield any significant difference in DPOAEs. However, in both these groups hearing loss was accompanied by a decrease in DPOAEs, specifically, at 2–6 kHz and 16 kHz. The results suggest that any decrease in DPOAEs seems to be related only to hearing loss and there is no additional effect from tinnitus.

## 1. Introduction

Tinnitus is the sense of perceiving sound when there is no such sound in the environment (reviewed in [1]). It may take the form of pure tones, noise, or other types of sounds. The exact mechanism of tinnitus generation is still unknown, but a possible explanation is that it is related to neural processing of information coming from damaged hair cells. Despite years of studies of this phenomenon, there is still no way of detecting it or measuring its severity without the cooperation of the patient [2]. One approach that has given some optimism in this regard is otoacoustic emissions (OAEs). OAEs are low-level sounds that originate from the cochlea and are detectable in the ear canal (reviewed in [3]). Their presence is directly linked to the normal functioning of outer hair cells, so it, therefore, seems likely that they are sensitive to such pathological conditions as tinnitus.

Tinnitus is, in most cases, associated with hearing loss [4]. Curiously, there are cases of people with profound hearing loss who experience a reduction in their tinnitus following cochlear implantation [5]. At the same time, there are also subjects who experience tinnitus even though their hearing is apparently normal [4].

OAEs are known to be even more sensitive to pathology than hearing thresholds determined by pure tone audiometry [6]. They have sometimes been recommended [7] as a tool to detect preclinical hearing loss (i.e., before a change can be seen in threshold). For these reasons, researchers have turned to OAEs as a possible marker for tinnitus—especially in cases where there is no detectable hearing loss. The results so far have been inconclusive. There are works that show changes in distortion product OAEs (DPOAEs) in subjects with normal hearing and tinnitus compared to controls. Some of them report decreases in DPOAEs of subjects with tinnitus [8,9,10,11,12], but interestingly others also report enhancement of DPOAEs at some frequencies [13,14,15]. There are also some works that have seen changes in DPOAEs only in a subset of subjects who have normal hearing and tinnitus (e.g., [16]); other authors are skeptical that the cochlea is even involved in tinnitus [17].

Delving deeper into the matter of tinnitus subjects with normal hearing, it is also worth noting that there are subjects who have very sensitive hearing (low thresholds) at very high frequencies (>8 kHz). In such cases—tinnitus subjects with normal hearing up to 8 kHz but higher thresholds at 10–16 kHz compared to controls—the findings are that they also have lower DPOAEs below 8 kHz [10]. So the question arises as to whether changes in DPOAEs might also be seen in tinnitus subjects who had similar thresholds to non-tinnitus controls right up to 16 kHz.

One relatively recent advance in DPOAE measurement is the possibility of acquiring responses to ultra-high frequencies (i.e., >8 kHz), as done by Dreisbach and Siegel [18]. There are now commercial systems capable of such measurements, but not many studies have used them to study tinnitus [19]. Indeed, there are relatively few studies of ultra-high DPOAEs at all [20,21,22,23].

The motivation for this study is that more information is needed on the relationship between tinnitus and OAEs. So far, reports relating the two are often contradictory. In fact, the relation between tinnitus and hearing loss is itself not clear—do OAEs diminish due to tinnitus or simply due to hearing loss alone, regardless of tinnitus? Another consideration is that there have not been many studies on ultra-high frequency DPOAEs for tinnitus subjects [10,11]. It is known that OAE levels can diminish before changes are visible in pure tone hearing thresholds, e.g., [24]. Do tinnitus subjects with normal hearing up to 16 kHz have weaker DPOAEs in the 8–16 kHz range? Can ultra-high frequency DPOAEs be used as a marker of preclinical hearing loss? The answers to these questions might be found by studying receiver operator characteristics (ROCs) in a population of tinnitus subjects, but to our knowledge, there are no studies that have used this approach.

The purpose of this work was to investigate DPOAEs up to 16 kHz in tinnitus subjects. The aim was to investigate whether weaker DPOAEs are due to tinnitus or due to hearing loss alone, irrespective of tinnitus. We used ROC analysis to evaluate whether DPOAEs, especially those at ultra-high frequencies, were diagnostic of hearing loss or tinnitus.

## 2. Materials and Methods

### 2.1. Participants

Participants consisted of adults without tinnitus (controls—C, 63 subjects, age 21–77 years, 31 females) and others that reported tinnitus (tinnitus positive—T, 55 subjects, age 18–74, 30 females).

All subjects had normal middle ear function verified by 226 Hz tympanometry (tympanometric peak pressure between –100 and +100 daPa and peak compensated static acoustic admittance of 0.3–1.3 mmhos). None had any known history of otologic disease.

There were 25 subjects with bilateral tinnitus and 30 with unilateral tinnitus (15 left). For the subjects with unilateral tinnitus, only one ear was included in the analyses. The data was also pooled regardless of the laterality of the subject’s tinnitus because recent studies have shown no difference in DPOAEs for tinnitus subjects with normal hearing who had unilateral or bilateral tinnitus [25]. All subjects reported that their tinnitus was tonal or like narrow-band noise. The duration of tinnitus was at least 6 months.

The subjects without tinnitus were chosen so as to audiometrically match a group of tinnitus subjects. Each group was divided into three subgroups according to the hearing threshold: better than 25 dB HL at all frequencies from 0.125 to 16 kHz (C16–20 ears, T16–13 ears); equal or better than 20 dB up to 8 kHz (C8–40 ears, T8–41 ears); and hearing impaired (Cim—24 ears, Tim—26 ears). The subsets were created by ears (not by subjects) since both ears of some subjects were used for the analyses (there were no cases of one person’s ears fitting two different subsets). In order to obtain groups of ears of similar size when considering only one ear for unilateral tinnitus, some ears of individuals without tinnitus also had to be excluded.

### 2.2. Procedures

The status of the ears of each subject was assessed using otoscopy (visual evaluation of the ear, mainly the tympanic membrane), pure tone audiometry, impedance audiometry (IA), distortion product otoacoustic emissions (DPOAEs), and tinnitus evaluation. Otoscopic examinations did not reveal any abnormalities.

Pure tone audiometry was performed using a Madsen Astera clinical audiometer (GN Otometrics, Taastrup, Denmark). Air conduction hearing thresholds were determined for 0.125 to 16 kHz using Sennheiser HDA-200 headphones. For normal hearing, a criterion of better than 25 dB HL was used.

IA in the form of tympanometry measurements was made using a Madsen Zodiac 901 impedance bridge (GN Otometrics). A standard test tone of 226 Hz was used.

DPOAEs were measured using the HearID system (Mimosa Acoustics Inc., Champaign, IL, USA) with an ER-10C probe (Etymotic Research, Elk Grove Village, IL, USA). DPOAEs were evoked by two tones at frequencies of F1 and F2, and responses were measured at a frequency of 2F1–F2. DPOAEs were measured at 8 selected frequencies for F2 of 1, 2, 4, 6, 8, 10, 12, and 16 kHz; the F2/F1 ratio was 1.2, and the stimulus levels were 65 and 55 dB SPL, respectively. The measurement settings used were the same as the default protocols of the HearID system; the only change was a different frequency arrangement, with an extension up to 16 kHz.

All OAE recordings were evaluated by standard OAE parameters—response levels, and signal to noise ratios (SNRs). Response levels were expressed in dB SPLs. SNR was evaluated as the difference in dB between the response level and the noise floor. As we wanted to compare results between subjects with normal and impaired hearing we included in the analysis all measured signals irrespectible of SNR criterion, (similarly to some other studies of similar design, e.g., [26]). Application of such criterion could introduce bias in ROC analysis.

### 2.3. Statistical Analysis

All analyses were made in Matlab (version 2018b, MathWorks, Natick, MA). For all measured parameters, the statistical significance of mean differences was evaluated using analysis of variance (ANOVA), and a *t*-test was used for pairwise comparisons. As a criterion of significance, a 95% confidence level (*p* < 0.05) was chosen. When conducting multiple comparisons, *p*-values were adjusted using the Benjamini and Hochberg [27] procedure to control false discovery rates.

Receiver operating characteristics (ROCs) and area under the ROC curve (AUC) were used to gauge the efficiency with which OAEs could diagnose the presence or absence of hearing loss or tinnitus. A ROC curve plots the relative proportion of hits (sensitivity) against the number of false alarms (1—specificity). Sensitivity is the likelihood of identifying an ear as impaired when hearing loss is present; specificity is the chance of identifying a normal-hearing ear as normal, and efficiency is the proportion of ears that are correctly identified. AUC ranges from 0.5 for a test with no diagnostic power to 1.0 for a test with perfect diagnostic ability. Some sources state that an AUC of 0.7 to 0.8 provides acceptable discrimination, 0.8 to 0.9—excellent, and more than 0.9—outstanding (e.g., [28]). The ROC analysis was summarized by AUCs and symmetry cutoff values (the point of highest sensitivity and specificity). The symmetry cutoff values can be used as a threshold for decision criteria (e.g., pass/refer).

## 3. Results

### 3.1. Pure Tone Thresholds

**Figure 1:**
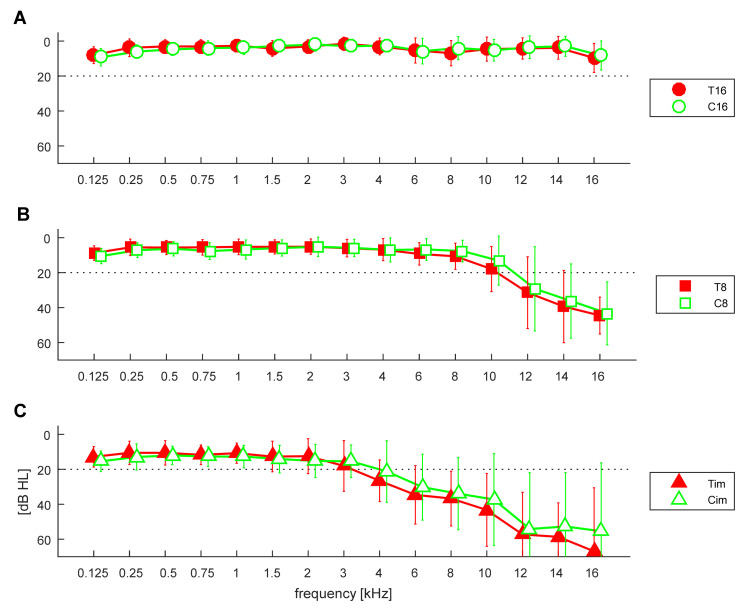
Average pure tone hearing thresholds in control groups (green—C) and in tinnitus patients (red—T) divided into three groups according to thresholds. (**A**) Thresholds better than 25 dB HL for frequencies up to 16 kHz—T16/C16. (**B**) Thresholds better than 25 dB HL up to 8 kHz—T8/C8. (**C**) Impaired—Tim/Cim. Whiskers indicate standard deviations.

The pure tone thresholds of described groups are shown in Figure 1. A repeated measures ANOVA (rmANOVA) was used to examine differences in pure tone thresholds as a function of group based on HL, tinnitus presence, and frequency (a repeated measure). It was found that there was a significant effect of HL on threshold [F(2,158) = 115.6, *p* < 0.001] but no effect of tinnitus [F(1,158) = 0.47, *p* = 0.49]. There was also a significant effect of frequency on threshold [F(14,2212) = 129.1, *p* < 0.001]. Additionally, there was an interaction effect of HL presence and frequency for threshold [F(28,2212) = 30.2, *p* < 0.001].

### 3.2. Comparison of Groups in Relation to Hearing Loss

**Figure 2:**
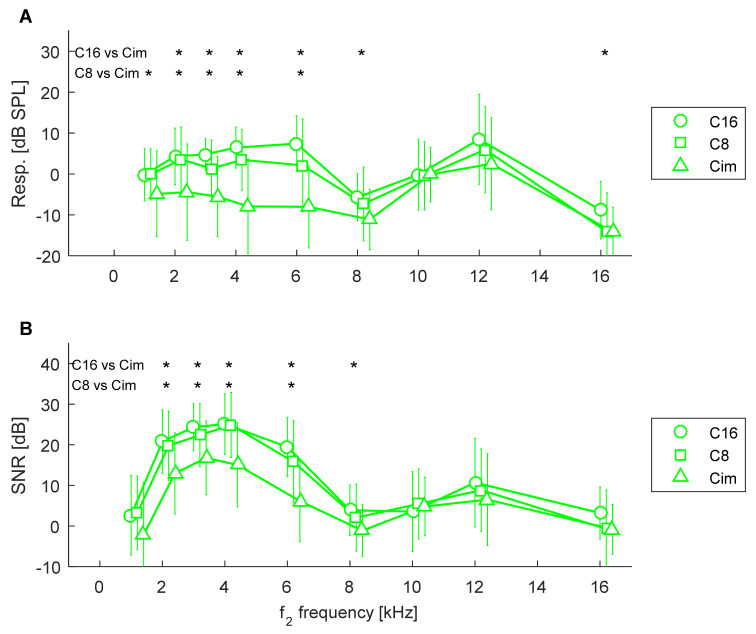
Average response levels (**A**) and SNRs (**B**) of DPOAEs from control groups for three PTA groups: C16, C8, and Cim. Whiskers indicate standard deviations. Asterisks mark significant differences between subgroups C16 vs. Cim, and C8 vs. Cim. There were no significant differences for C16 vs. C8.

First, we checked whether ultra-high frequency DPOAEs were affected by hearing loss in ears without tinnitus (using the control groups C16, C8, Cim). The response levels and SNRs of DPOAEs for C ears (without tinnitus) are shown in Figure 2. It can be seen that there are only small differences between groups C16 and C8 while there are more pronounced differences between both C16 and Cim, and C8 and Cim, mostly in the 2–6 kHz range.

An rmANOVA was used to examine differences in response levels and SNRs as a function of HL and frequency (a repeated measure). It showed a significant effect of HL on response level [F(2,81) = 15.9, *p* < 0.001] and on SNR [F(2,81) = 10.0, *p* < 0.001]. As expected, there was also a significant effect of frequency on response level (since DPOAEs have different properties across frequencies) [F(8,648) = 38.3, *p* < 0.001] and on SNR [F(8,648) = 94.9, *p* < 0.001]. Additionally, there was an interaction effect of HL presence and frequency for both response level [F(16,648) = 3.2, *p* < 0.001] and SNR [F(16,648) = 2.3, *p* = 0.003].

Pairwise comparisons for response levels at different frequencies showed significant differences between C16 and Cim for frequencies of 2–8 kHz and 16 kHz, and between C8 and Cim for frequencies of 1–6 kHz (marked by asterisks in Figure 2A).

Likewise, for SNR, pairwise comparisons of SNRs at different frequencies showed significant differences between C16 and Cim for frequencies of 2–8 kHz, and between C8 and Cim for frequencies of 2–6 kHz (marked by asterisks in Figure 2B).

### 3.3. Comparison between Controls and Tinnitus Subjects

**Figure 3:**
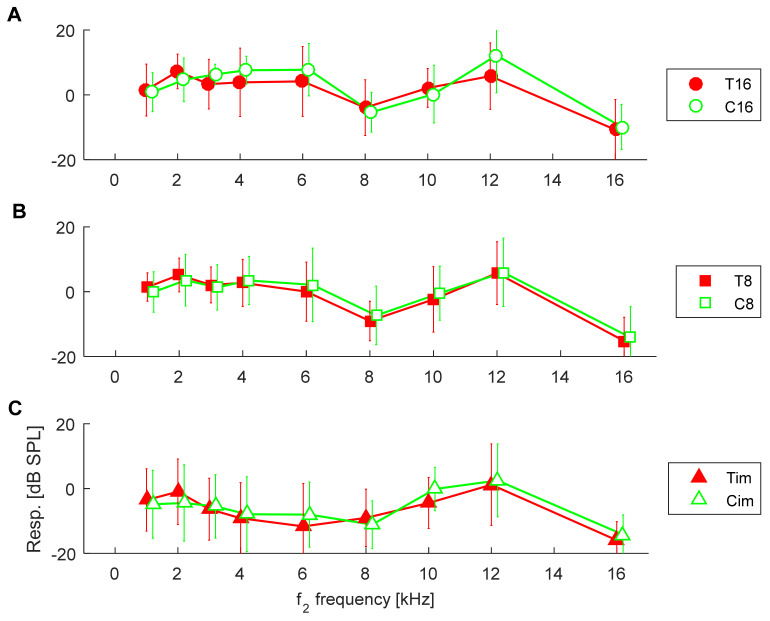
Average response levels of DPOAEs in control groups (green) and in tinnitus patients (red) according to the threshold groupings (**A**–**C**) shown in Figure 1. Whiskers indicate standard deviations. There were no significant differences between the tinnitus and control groups.

**Figure 4:**
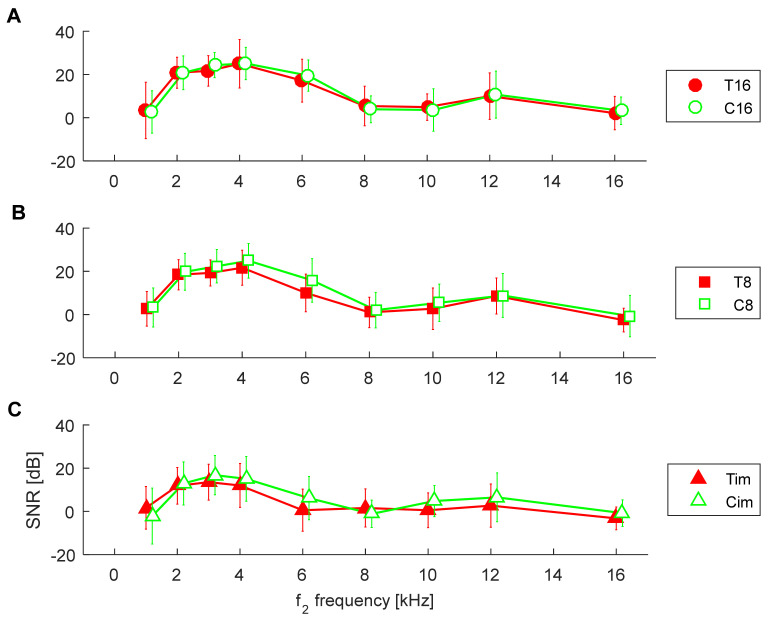
Average SNRs of DPOAEs in control groups (green) and in tinnitus patients (red) according to the threshold groupings (**A**–**C**) shown in Figure 1. Whiskers indicate standard deviations. There were no significant differences between the tinnitus and control groups.

In order to try to isolate the effect of tinnitus from the effect of HL, we now compare, according to their hearing thresholds, the control groups (C16, C8, Cim) with the matching tinnitus groups (T16, T8, Tim), as shown in Figure 1. The corresponding DPOAE response levels for both groups are shown in Figure 3, while SNRs are shown in Figure 4. In these latter two figures, the control groups C are the same as in Figure 2, and it can be seen that the DPOAE properties are virtually the same for both the tinnitus subjects and the control subjects.

When comparing the T16 group with the C16 group, an rmANOVA showed no significant effect of tinnitus on response level [F(1,31) = 0.06, *p* = 0.8], as shown in Figure 3A, or on SNR [F(1,31) = 0.05, *p* = 0.8], as shown in Figure 4A. As expected, there was a significant effect of frequency on response level [F(8,248) = 21.0, *p* < 0.001] and on SNR [F(8,248) = 45.2, *p* < 0.001]. Additionally, there was no interaction between tinnitus presence and frequency for both response level [F(8,248) = 0.99, *p* = 0.43] and SNR [F(8,248) = 0.29, *p* = 0.9].

When comparing the T8 group with the C8 group, an rmANOVA showed no significant effect of tinnitus on response level [F(1,79) = 0.20, *p* = 0.65], as shown in Figure 3B, but there was an effect on SNR [F(1,79) = 4.48, *p* = 0.037], as shown in Figure 4B. However, pairwise comparisons did not yield significant result at any frequency. As expected, there was a significant effect of frequency on response level [F(8,632) = 64.8, *p* < 0.001] and on SNR [F(8,632) = 129.9, *p* < 0.001]. Additionally, there was no interaction between tinnitus presence and frequency for both response level [F(8,632) = 0.77, *p* = 0.62] and SNR [F(8,632) = 1.12, *p* = 0.34].

When comparing the Tim group with the Cim group, an rmANOVA showed no significant effect of tinnitus on response level [F(1,48) = 0.17, *p* = 0.68]—see Figure 3C—or on SNR [F(1,48) = 2.03, *p* = 0.16]—see Figure 4C. As expected, there was a significant effect of frequency on response level [F(8,384) = 18.2, *p* < 0.001] and on SNR [F(8,384) = 29.9, *p* < 0.001]. Additionally, there was no interaction between tinnitus presence and frequency for both response level [F(8,384) = 1.15, *p* = 0.33] and SNR [F(8,384) = 1.81, *p* = 0.074].

### 3.4. Discrimination of Hearing Loss

We wanted to check if ultra-high DPOAEs can discriminate HL. To do this, an ROC analysis was performed between groups having different hearing thresholds, that is, C16 vs. C8, C16 vs. Cim, and C8 vs. Cim. The results of this analysis are summarized in Table 1 and show AUCs and symmetry cutoff values for response levels at different frequencies. The C16 vs. C8 comparison (last column) shows acceptable discrimination (AUC = 0.7) only for F2 = 16 kHz (in the tables, ROC values higher than 0.70 are highlighted in red). The C16 vs. Cim comparison showed acceptable discrimination for 2 and 8 kHz, and excellent discrimination for the 3–6 kHz range. The C8 vs. Cim comparison showed acceptable discrimination over the 2–6 kHz range.

A similar analysis for SNRs yielded slightly worse results (Table 2). The C16 vs. C8 comparison did not show acceptable discrimination for any frequency. The C16 vs. Cim comparison showed acceptable discrimination (AUC > 0.70) for 2, 3, 4, and 8 kHz, and excellent discrimination (AUC = 0.86) for 6 kHz. The C8 vs. Cim comparison showed acceptable discrimination for the 2–6 kHz range (AUC > 0.70).

### 3.5. Discrimination of Tinnitus

Since the main idea of this study was to check whether DPOAEs might be sensitive to tinnitus only (regardless of HL), the ROC analysis was performed using DPOAEs from groups that had matched hearing thresholds: C16 vs. T16, C8 vs. T8, and Cim vs. Tim. The results of this analysis for DPOAE response levels are summarized in Table 3. As this table shows, the AUC did not reach 0.7 (acceptable discrimination) at any frequency. Very similar results were obtained for SNRs (not shown).

### 3.6. Examples of Criteria and Their Performance

When DPOAEs are used as a functional test, certain criteria are employed to determine whether a signal is present. Often the criteria are certain response levels or SNRs that should be met for particular frequencies (e.g., three of six identified frequencies). Such an approach was used here. Table 4 lists six criteria, based on a combination of several frequencies, which were used to evaluate the sensitivity, specificity, and efficiency with which DPOAEs could detect HL. The criteria we selected are based on the six best frequencies shown in Table 1—that is, 2, 3, 4, 6, 8, and 16 kHz—and use the symmetry cutoff values as set out in the column headed C16 vs. Cim. The best performance, as evaluated by efficiency, was when 3 or 4 of the DPOAE frequencies (out of 6) which exceeded the symmetry cutoff value were used.

## 4. Discussion

This work has shown that when tinnitus subjects are matched to a control group with similar pure tone audiometry results, there seems to be no difference in their DPOAEs. At the same time, we have duplicated the finding that DPOAEs are reduced when there is mild hearing loss. An additional result of interest is that ultra-high frequency DPOAEs (at F2 = 16 kHz) may have the potential to detect hearing loss at this frequency. Surprisingly, DPOAEs at F2 = 10 or 12 kHz do not seem to provide this capability.

### 4.1. Relationship of DPOAEs to Tinnitus

Some earlier studies have claimed that there is a difference in DPOAEs between subjects with tinnitus and controls, both with normal hearing thresholds (e.g., [8,9,11,12]). Here we found no such dependence. The discrepancy might be related to the underlying methodology, i.e., what we understand to be a normal hearing threshold [29]. If one takes as criterion better than 25 dB HL, it is possible to have two subjects, one with a 0 dB threshold and another with a 20 dB threshold—a difference of as much as 20 dB. In the present study, we constructed our dataset to ensure that there was no significant difference between the hearing thresholds of the tinnitus group and the control group.

The present results support some recent studies using various techniques, which seem to point to some extra-cochlear source of tinnitus (e.g., [30]). In this context, our work is probably the first to explore the relationship between ultra-high frequency hearing thresholds and DPOAEs. We set out to investigate whether ultra-high frequency DPOAEs might be suitable as a marker of preclinical changes that cannot be observed with pure tone audiometry. As it turned out, we did not succeed in uncovering any such relationship.

### 4.2. Relationship between DPOAEs and HL

As discussed above, we did not succeed in showing any effect of tinnitus on DPOAEs. However, we did find some potential for using ultra-high frequency DPOAEs to detect changes in hearing. This aspect looks promising, but at the same time should be treated with caution as the reliability of DPOAEs at frequencies above 6 kHz is worse than at lower frequencies (e.g., [31,32]). This reliability issue might also explain why, at 10–12 kHz, we saw no difference in DPOAEs between groups that had different hearing thresholds.

Looking at the general properties of DPOAEs, the minimum level at 8 kHz and maximum at 12 kHz seem to relate to the characteristics of OAEs and the middle ear transfer function. These features have also been noted in measurements using different systems [31,33].

The ROC results obtained here are similar to previous studies which have shown the best performance of DPOAEs in the 2–8 kHz range (e.g., [26,34]). The quite promising result for 16 kHz cannot be compared as there appears to be no other ROC data at this frequency.

### 4.3. Limitations

It should be noted that there are reports that the ER-10C probe used here for DPOAE assessment tends to generate artifacts at frequencies greater than 8 kHz [35]. Indeed, the poor discrimination shown here between groups at frequencies above 8 kHz may be indirect proof of such problems. From the analyses presented here, it may appear that DPOAEs at 16 kHz are excluded from the influence of artifacts. Nevertheless, this may also be a result of too small a dataset. Therefore, the overall results presented here for frequencies above 8 kHz need to be treated with caution.

### 4.4. Thoughts on the Usefulness of OAEs

This study tends to support some other recent studies that have put a question mark over previous findings. For example, Riga et al. [36] showed that, in various studies of tinnitus subjects, suppression of DPOAEs by contralateral stimulation (CAS) differs so much in design, instrumentation, protocol, and subjects that it is virtually impossible to compare them. Some other confounding factors which are relatively rarely discussed, such as differences between results obtained using different equipment [37] and certain calibration issues, e.g., [35], also need to be considered. Certainly, if OAEs are to be useful diagnostically, we need to first look critically at some earlier results. Furthermore, OAEs need to give either objective information that aligns with other methods, especially pure tone audiometry, or give precise and certain preclinical information (for example, a prediction of a change in high-frequency pure tone audiometry that is later verified). More studies of ROC analysis on DPOAEs are needed because so far there has only been a limited number. The diverse findings regarding tinnitus (e.g., [9,10,11,12]) imply that DPOAEs are unlikely to form the basis of reproducible findings, and, more to the point, appear to indicate that we are far away from using them to provide a clinical basis for diagnosing tinnitus.

## 5. Conclusions

The present study shows that when tinnitus subjects were matched with a control group having similar hearing thresholds there were no apparent differences in DPOAEs, i.e., the decrease in DPOAEs seems to be related only to hearing loss and there was no additional effect of tinnitus. This would suggest that DPOAEs are not helpful in detecting tinnitus. Nevertheless, in agreement with previous studies, DPOAEs can be used to differentiate hearing loss in the 2–8 kHz range. Furthermore, DPOAEs at 16 kHz have the potential to detect preclinical decreases in the hearing threshold.

The results here suggest that tinnitus generation is not directly related to cochlear mechanics and that the tinnitus sensation is probably generated further along the auditory pathway. This is in line with some recent imaging studies that were able to differentiate tinnitus subjects from controls based on activation of certain brain regions [38,39].

## Figures and Tables

**Table 1:** ROC analysis when DPOAE response levels (at each frequency shown) were used to discriminate hearing loss between groups C16, C8, and Cim. AUCs and symmetry cutoff values shown.

	AUC			Symmetry Cutoff Value		
F [kHz]	C16 vs. Cim	C8 vs. Cim	C16 vs. C8	C16 vs. Cim	C8 vs. Cim	C16 vs. C8
1	0.63	0.63	0.52	−0.9	−4.1	1.3
2	0.72	0.73	0.51	2	0.6	8.3
3	0.86	0.74	0.63	2.1	2.2	4.4
4	0.87	0.79	0.62	0.5	−0.3	5.8
6	0.87	0.75	0.60	1.6	−3.2	4.1
8	0.71	0.64	0.55	−8	−9.2	−5
10	0.50	0.48	0.51	−0.1	−0.1	−0.1
12	0.63	0.60	0.53	5.7	0.5	5.7
16	0.70	0.48	0.70	−12.6	−12.7	−12.6

**Table 2:** ROC analysis when DPOAE SNRs (at each frequency shown) were used to discriminate hearing loss between groups C16, C8, and Cim. AUCs and symmetry cutoff values shown.

	AUC			Symmetry Cutoff Value		
F [kHz]	C16 vs. Cim	C8 vs. Cim	C16 vs. C8	C16 vs. Cim	C8 vs. Cim	C16 vs. C8
1	0.66	0.66	0.48	−2.2	−2.1	1.9
2	0.74	0.72	0.52	17.9	17.4	25.6
3	0.79	0.70	0.55	23.3	22.9	26.2
4	0.78	0.77	0.51	20.5	21.2	26.1
6	0.86	0.75	0.60	12.5	11.9	14.4
8	0.73	0.62	0.59	3.2	1	3.2
10	0.44	0.53	0.42	8.3	7.5	8.3
12	0.58	0.56	0.51	1.8	6.5	10.4
16	0.64	0.47	0.68	−1.4	−0.6	2.3

**Table 3:** ROC analysis when DPOAE response levels (at each frequency shown) used to discriminate tinnitus between groups C16, C8, Cim and T16, T8, Tim. AUCs and symmetry cutoff values shown.

	AUC			Symmetry Cutoff Value		
F [kHz]	C16 vs. T16	C8 vs. T8	Cim vs. Tim	C16 vs. T16	C8 vs. T8	Cim vs. Tim
1	0.44	0.42	0.46	−1.6	4.1	−5.4
2	0.39	0.48	0.43	9.5	5.1	−1.7
3	0.54	0.53	0.54	3	2.6	−0.8
4	0.56	0.54	0.53	6.7	4.4	−6.4
6	0.57	0.59	0.57	6.4	6.5	−14.6
8	0.38	0.58	0.42	−8	−9.2	−7.6
10	0.42	0.56	0.69	3.6	−2.6	−1.3
12	0.60	0.51	0.52	5.7	10.3	5.5
16	0.56	0.55	0.60	−8.8	−15.7	−15.6

**Table 4:** Sensitivity, specificity, and efficiency for groups C16 and Cim based on various numbers of six best symmetry cutoff values (for frequencies 2, 3, 4, 6, 8, and 16 kHz).

No. of Frequencies	Sensitivity (%)	Specificity (%)	Efficiency (%)
1 of 6	25	100	59
2 of 6	67	95	80
3 of 6	79	95	86
4 of 6	83	85	84
5 of 6	88	50	70
6 of 6	100	15	61

## Data Availability

The data underlying this article will be shared on reasonable request to the corresponding authors.

## References

[1] Haider H.F., Hoare D.J., Ribeiro S.F., Ribeiro D., Caria H., Trigueiros N., Borrego L.M., Szczepek A.J., Papoila A.L., Elarbed A. (2021). Evidence for biological markers of tinnitus: A systematic review. Prog. Brain Res..

[2] Jackson R., Vijendren A., Phillips J. (2019). Objective Measures of Tinnitus: A Systematic Review. Otol. Neurotol..

[3] Probst R., Lonsbury-Martin B.L., Martin G.K. (1991). A review of otoacoustic emissions. J. Acoust. Soc. Am..

[4] Henry J.A., Roberts L.E., Caspary D.M., Theodoroff S.M., Salvi R.J. (2014). Underlying mechanisms of tinnitus: Review and clinical implications. J. Am. Acad. Audiol..

[5] Olze H., Szczepek A.J., Haupt H., Zirke N., Graebel S., Mazurek B. (2012). The impact of cochlear implantation on tinnitus, stress and quality of life in postlingually deafened patients. Audiol. Neurootol..

[6] Marshall L., Lapsley Miller J.A., Heller L.M. (2001). Distortion-Product Otoacoustic Emissions as a Screening Tool for Noise-Induced Hearing Loss. Noise Health.

[7] De PCampos U., Sanches S.G., Hatzopoulos S., Carvallo R.M., Kochanek K., Skarżyński H. (2012). Alteration of distortion product otoacoustic emission input/output functions in subjects with a previous history of middle ear dysfunction. Med. Sci. Monit..

[8] Ozimek E., Wicher A., Szyfter W., Szymiec E. (2006). Distortion product otoacoustic emission (DPOAE) in tinnitus patients. J. Acoust. Soc. Am..

[9] Ami M., Abdullah A., Awang M.A., Liyab B., Saim L. (2008). Relation of distortion product otoacoustic emission with tinnitus. Laryngoscope.

[10] Fabijańska A., Smurzyński J., Hatzopoulos S., Kochanek K., Bartnik G., Raj-Koziak D., Mazzoli M., Skarżyński P.H., Jędrzejczak W.W., Szkiełkowska A. (2012). The relationship between distortion product otoacoustic emissions and extended high-frequency audiometry in tinnitus patients. Part 1: Normally hearing patients with unilateral tinnitus. Med. Sci. Monit..

[11] Park J.P., Lim H.W., Shim B.S., Kim T.S., Chung J.W., Yoon T.H., Park H.J. (2013). Interaural differences of distortion product otoacoustic emission amplitudes in patients with unilateral tinnitus. Otolaryngol. Head Neck Surg..

[12] Modh D., Katarkar A., Alam N., Jain A., Shah P. (2014). Relation of distortion product otoacoustic emission and tinnitus in normal hearing patients: A pilot study. Noise Health.

[13] Gouveris H., Maurer J., Mann W. (2005). DPOAE-grams in patients with acute tonal tinnitus. Otolaryngol. Head Neck Surg..

[14] Sztuka A., Pospiech L., Gawron W., Dudek K. (2010). DPOAE in estimation of the function of the cochlea in tinnitus patients with normal hearing. Auris Nasus Larynx.

[15] Husain F.T. (2013). Effect of tinnitus on distortion product otoacoustic emissions varies with hearing loss. Am. J. Audiol..

[16] Onishi E.T., Fukuda Y., Suzuki F.A. (2004). Distortion product otoacoustic emissions in tinnitus patients. Int. Tinnitus J..

[17] Serra L., Novanta G., Sampaio A.L., Oliveira C.A., Granjeiro R., Braga S.C. (2015). The study of otoacoustic emissions and the suppression of otoacoustic emissions in subjects with tinnitus and normal hearing: An insight to tinnitus etiology. Int. Arch. Otorhinolaryngol..

[18] Dreisbach L.E., Siegel J.H. (2001). Distortion-product otoacoustic emissions measured at high frequencies in humans. J. Acoust. Soc. Am..

[19] Sanchez T.G., Moraes F., Casseb J., Cota J., Freire K., Roberts L.E. (2016). Tinnitus is associated with reduced sound level tolerance in adolescents with normal audiograms and otoacoustic emissions. Sci. Rep..

[20] Dunckley K.T., Dreisbach L.E. (2004). Gender effects on high frequency distortion product otoacoustic emissions in humans. Ear Hear..

[21] Kei J., Brazel B., Crebbin K., Richards A., Willeston N. (2007). High frequency distortion product otoacoustic emissions in children with and without middle ear dysfunction. Int. J. Pediatr. Otorhinolaryngol..

[22] Trzaskowski B., Jedrzejczak W.W., Pilka E., Cieslicka M., Skarzynski H. (2014). Otoacoustic emissions before and after listening to music on a personal player. Med. Sci. Monit..

[23] Dreisbach L., Zettner E., Chang Liu M., Fernhoff C.M., MacPhee I., Boothroyd A. (2018). High-Frequency Distortion-Product Otoacoustic Emission Repeatability in a Patient Population. Ear Hear..

[24] Arnold D.J., Lonsbury-Martin B.L., Martin G.K. (1999). High-frequency hearing influences lower-frequency distortion-product otoacoustic emissions. Arch. Otolaryngol. Head Neck Surg..

[25] Zagólski O., Stręk P. (2017). Comparison of characteristics observed in tinnitus patients with unilateral vs bilateral symptoms, with both normal hearing threshold and distortion-product otoacoustic emissions. Acta Otolaryngol..

[26] Blankenship C.M., Hunter L.L., Keefe D.H., Feeney M.P., Brown D.K., McCune A., Fitzpatrick D.F., Lin L. (2018). Optimizing Clinical Interpretation of Distortion Product Otoacoustic Emissions in Infants. Ear Hear..

[27] Benjamini Y., Hochberg Y. (1995). Controlling the false discovery rate—Practical and powerful approach to multiple testing. J. R. Stat. Soc. B.

[28] Mandrekar J.N. (2010). Receiver operating characteristic curve in diagnostic test assessment. J. Thorac. Oncol..

[29] Pienkowski M. (2017). On the Etiology of Listening Difficulties in Noise Despite Clinically Normal Audiograms. Ear Hear..

[30] Guest H., Munro K.J., Prendergast G., Howe S., Plack C.J. (2007). Tinnitus with a normal audiogram: Relation to noise exposure but no evidence for cochlear synaptopathy. Hear. Res..

[31] Dreisbach L.E., Long K.M., Lees S.E. (2006). Repeatability of high-frequency distortion-product otoacoustic emissions in normal-hearing adults. Ear Hear..

[32] Pilka E., Jedrzejczak W.W., Trzaskowski B., Skarzyński H. (2014). Variability of distortion product otoacoustic emissions at 10, 12 and 16 kHz: A preliminary study. J. Hear. Sci..

[33] Pilka E., Jedrzejczak W.W., Olszewski L., Skarzynski H. (2016). High-frequency distortion product otoacoustic emissions measured by two systems: An example of a subject with normal hearing. J. Hear. Sci..

[34] Torre P., Cruickshanks K.J., Nondahl D.M., Wiley T.L. (2003). Distortion product otoacoustic emission response characteristics in older adults. Ear Hear..

[35] Charaziak K.K., Shera C.A. (2017). Compensating for ear-canal acoustics when measuring otoacoustic emissions. J. Acoust. Soc. Am..

[36] Riga M., Katotomichelakis M., Danielides V. (2015). The potential role of the medial olivocochlear bundle in the generation of tinnitus: Controversies and weaknesses in the existing clinical studies. Otol. Neurotol..

[37] Jedrzejczak W.W., Gos E., Pilka E., Skarzynski P.H., Skarzynski H., Hatzopoulos S. (2021). Pitfalls in the Detection of Hearing Loss via Otoacoustic Emissions. Appl. Sci..

[38] Zimmerman B.J., Abraham I., Schmidt S.A., Baryshnikov Y., Husain F.T. (2018). Dissociating tinnitus patients from healthy controls using resting-state cyclicity analysis and clustering. Netw. Neurosci..

[39] Han L., Na Z., Chunli L., Yuchen C., Pengfei Z., Hao W., Xu C., Peng Z., Zheng W., Zhenghan Y. (2019). Baseline Functional Connectivity Features of Neural Network Nodes Can Predict Improvement After Sound Therapy Through Adjusted Narrow Band Noise in Tinnitus Patients. Front. Neurosci..

